# Effect of Initial Texture on Deformation Mechanisms and Texture Evolution in a Mg–9 wt.% Ag Alloy During High-Temperature Plane Strain Compression

**DOI:** 10.3390/ma19204319

**Published:** 2026-10-14

**Authors:** Jimin Yun, Yebeen Ji, Kibeom Kim, Sanggu Choi, Tae Hee Lee, Kwonhoo Kim

**Affiliations:** 1 Department of Marine Design Convergence Engineering, Pukyong National University, 45 Yongso-ro, Nam-gu, Busan 48513, Republic of Korea; dbswlals333@naver.com (J.Y.); choisanggu123@gmail.com (S.C.); 2 Department of Advanced Materials Science, Graduate School of Frontier Sciences, The University of Tokyo, 5-1-5, Kashiwanoha, Kashiwa-shi 277-8561, Chiba, Japan; wldpqls@gmail.com (Y.J.); rlarlqja0874@gmail.com (K.K.); 3 Vehicle Performance Optimization R&D Center Jeonnam Branch, Korea Automotive Technology Institute, 46, F1-ro, Samho-eup, Yeongam-gun, Jeonnam-Gwangju 58463, Republic of Korea; 4 Department of Metallurgical Engineering, Pukyong National University, 45 Yongso-ro, Nam-gu, Busan 48513, Republic of Korea

**Keywords:** Mg-Ag alloy, plane strain compression, initial texture, basal texture, dynamic recrystallization

## Abstract

The effect of initial texture on slip activity, dynamic recrystallization, and basal texture development was investigated in a Mg–9 wt.% Ag alloy during high-temperature plane strain compression. Three sets of specimens with different initial compression planes were machined from a hot-rolled plate and compressed at 723 K and a strain rate of 5.0 × 10^−2^ s^−1^ up to a true strain of −1.0. Microstructure and texture analyses revealed that the initial texture strongly affected the subsequent texture evolution and microstructural development. Sample A, which initially exhibited a strong basal texture, retained and intensified the basal component during deformation, whereas Sample B maintained a transverse direction texture component and showed the lowest flow stress, the highest kernel average misorientation, the lowest dynamically recrystallized fraction, and the retention of coarse unrecrystallized grains. These features suggest that prismatic slip was geometrically favored at the early stage of deformation, resulting in enhanced intragranular deformation and delayed rotation toward the stable basal texture. In contrast, Sample C initially exhibited a rolling direction texture component but rapidly evolved toward a strong basal texture, particularly between true strains of −0.7 and −1.0. Although dynamic recrystallization contributed to grain refinement and local orientation scattering, the final texture evolution was governed mainly by slip induced lattice rotation rather than recrystallization induced texture randomization. These results demonstrate that, although Ag addition can modify flow and recrystallization behavior, basal texture development remains dominant when slip-induced lattice rotation is strongly favored by the imposed plane strain compression geometry.

## 1. Introduction

Magnesium (Mg) and its alloys have attracted continuous attention as lightweight structural materials and biomaterials owing to their low density, high specific strength, excellent damping capacity, and biocompatibility [1,2,3]. In particular, Mg alloys have been considered promising candidate materials for weight reduction in aerospace, automotive, and mobility applications [4,5]. They have also been proposed for biomedical applications, such as orthopedic implants, because of their biodegradability and elastic modulus comparable to that of natural bone [2,6,7]. However, Mg alloys generally exhibit poor formability at room temperature owing to the limited number of active slip systems associated with their hexagonal close-packed (HCP) crystal structure. In addition, thermomechanical processing, such as rolling and compression, often produces a strong basal texture, resulting in pronounced plastic anisotropy and further limiting the formability of Mg alloys [8]. During conventional rolling or compressive deformation, the c-axis of Mg alloys tend to align along specific directions, typically toward the normal direction (ND) in rolled sheets, resulting in pronounced differences in yielding behavior and ductility depending on the deformation direction. Although such a basal texture can favor the activation of basal <a> slip, it makes deformation along the c-axis difficult to accommodate, thereby requiring the activation of non-basal slip systems or deformation twinning. Therefore, understanding the development behavior of basal texture and the deformation mechanisms controlling it is essential for improving the formability and reducing the plastic anisotropy of Mg alloys [8,9].

Under hot processing conditions, the difference in critical resolved shear stress (CRSS) between basal and non-basal slip decreases, thereby increasing the activation potential of non-basal slip systems such as prismatic <a> slip and pyramidal <c+a> slip [10,11]. The relative activity of these slip systems depends on the initial texture, deformation temperature, strain rate, stress state, and dynamic recrystallization (DRX) behavior. Plane strain compression provides a constrained deformation condition similar to rolling, while allowing the systematic analysis of texture evolution as a function of the initial texture orientation and specimen direction. Therefore, investigating the effects of the initial texture on slip activation, lattice rotation, and DRX behavior during high-temperature plane strain compression is of significant importance for understanding texture control mechanism during the hot processing of Mg alloys.

For AZ-series Mg alloys such as AZ31(MgAl3Zn1), AZ80(MgAl8Mn0.5Zn0.1), and AZ91(MgAl9Zn1), research has been conducted on the development of microstructure and texture depending on the initial texture during high-temperature plane strain compression [8,12,13,14]. Previous studies have shown that, even under the same temperature and strain rate conditions, the geometrical favorability for the activation of basal, prismatic, and pyramidal slip systems varies depending on the initial texture and the relative orientation between the c-axis and the compression axis. Accordingly, the texture components formed after deformation and the associated DRX behavior are altered. For example, it has been reported that, for certain initial orientations, the basal texture is maintained or strengthened as a stable component, whereas orientations favorable for prismatic slip can retain a metastable texture component in which the c-axis is associated with the Transverse Direction (TD) [8,13,14]. In addition, under conditions where pyramidal <c+a> slip is relatively activated, behavior related to the tilting of basal poles or texture weakening may occur. These results indicate that texture evolution during high-temperature deformation of Mg alloys is not determined solely by the strain, but is governed by the coupled effects of active slip systems and recrystallization behavior, both of which are strongly influenced by the initial texture.

Mg–Ag alloys have attracted attention as biomaterials and functional Mg alloys owing to the antibacterial activity of Ag and its relatively high solubility in the Mg matrix. Ag addition can influence the mechanical properties, corrosion behavior, and biological functionality of Mg alloys. Previous studies have reported grain refinement and changes in mechanical and corrosion properties of Mg–Ag alloys through extrusion, heat treatment, and severe plastic deformation [15,16,17,18]. However, these studies have primarily focused on biocompatibility, corrosion behavior, precipitation, and mechanical properties, whereas the influence of initial texture on slip activity, dynamic recrystallization, and texture evolution during high-temperature deformation remains insufficiently understood. Although the relatively high cost and density of Ag should be considered when evaluating the practical applicability of Mg–Ag alloys, investigating their deformation and texture evolution behavior remains relevant for understanding the effects of initial texture during hot working.

Therefore, in this study, high-temperature plane strain compression tests were conducted on a Mg–9 wt.% Ag alloy at 723 K to investigate the effects of initial texture orientation on microstructural development, slip activity, dynamic recrystallization, and subsequent texture evolution. Three specimens with different initial texture orientations, designated as Samples A, B, and C, were prepared from a hot-rolled plate. Microstructural and crystallographic characteristics were examined at different strain levels using electron backscatter diffraction (EBSD), pole figures, Schmid factor analysis, kernel average misorientation (KAM), and grain orientation spread (GOS). Particular attention was given to the relative contributions of basal and prismatic slip and their relationships with dynamic recovery, recrystallization, and basal texture development. The observed behavior was also discussed in comparison with previous findings for conventional AZ series Mg alloys.

## 2. Materials and Methods

The Mg–9 wt.% Ag alloy ingot used in this study was prepared by casting high purity magnesium (99%) and silver (99%) (Suzhou Haichuan Rare Metal Products Co., Suzhou, China). The chemical composition of the alloy was analyzed using X-ray fluorescence spectroscopy (XRF; XRF-1800, SHIMADZU, Tokyo, Japan). For this analysis, specimens with dimensions of 10 mm × 10 mm × 4 mm were cut and machined from the cast ingot. The measured chemical composition is presented in Table 1.

A cylindrical ingot with a diameter of 100 mm and a thickness of 13.4 mm was heated at 673 K for 1 h. It was subjected to repeated rolling until the total thickness reduction reached 25%. To prevent heat loss and crack formation during rolling, the reduction per pass was limited to 1–3%. After each pass, the specimen was reheated for 10 min to maintain the temperature at 673 K. After the target reduction was reached, the rolled specimen was water quenched to suppress additional microstructural changes.

**Figure 1:**
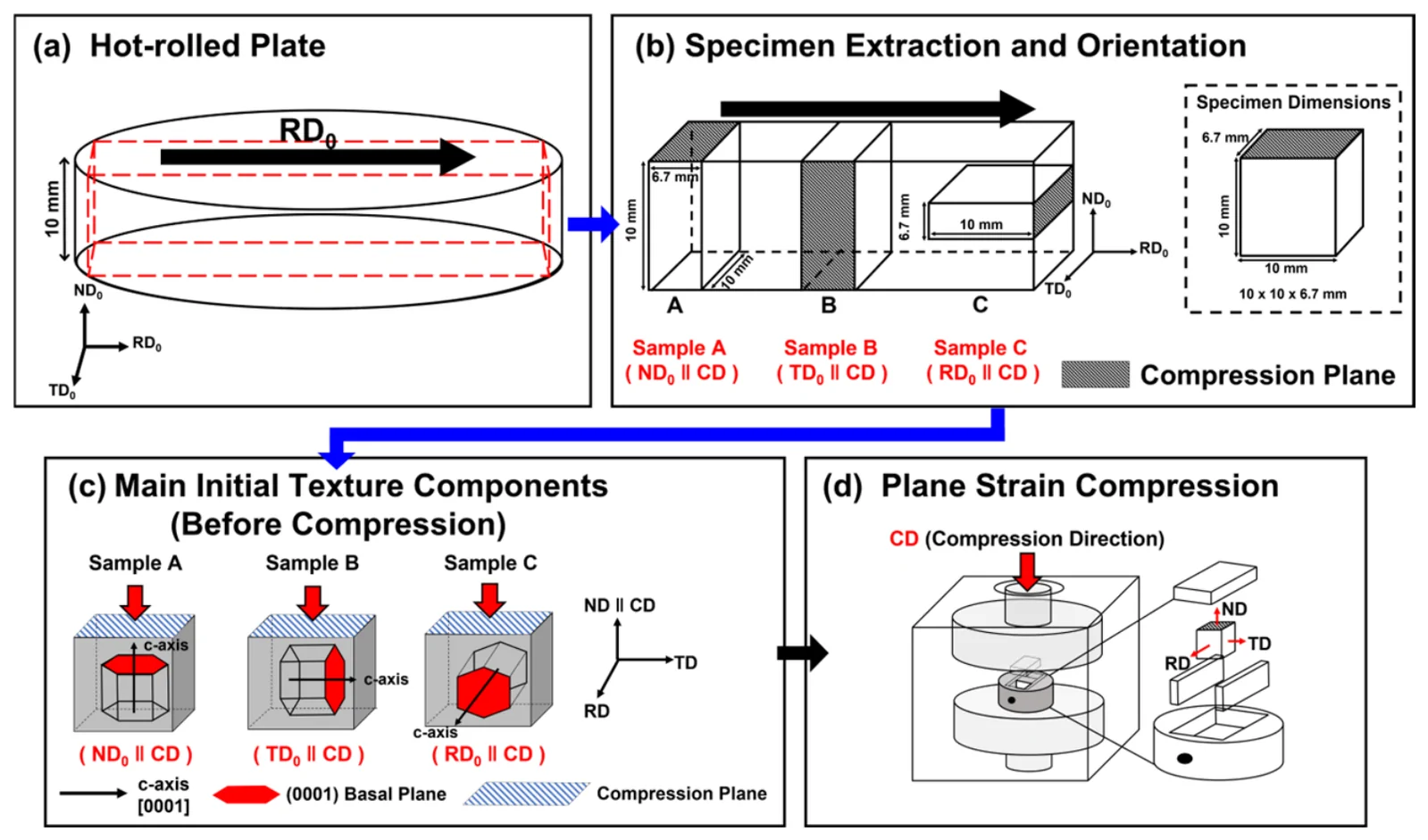
Schematic illustration of specimen preparation, initial texture orientations, and plane strain compression (PSC). Samples A, B, and C were designated according to the alignment of the original normal (ND_0_), transverse (TD_0_), and rolling (RD_0_) directions with the compression direction (CD), respectively.

As shown in Figure 1, specimens with dimensions of 10 × 10 × 6.7 mm^3^ were machined from the hot-rolled plate with different orientations relative to the original rolling coordinates. The specimens were designated as Sample A (ND_0_ ∥ CD), Sample B (TD_0_ ∥ CD), and Sample C (RD_0_ ∥ CD), according to the alignment of the original rolling coordinates with the compression direction (CD). Here, RD_0_, TD_0_, and ND_0_ denote the rolling, transverse, and normal directions of the original hot-rolled plate, respectively, whereas RD, TD, and ND represent the specimen coordinate system used during Plane Strain Compression (PSC). The corresponding main initial texture components are schematically illustrated in Figure 1c. Before compression testing, the specimens were homogenized at 773 K for 1 h to make a single-phase structure in the Mg–Ag alloy. To prevent oxidation during heat treatment, the specimens were vacuum sealed in quartz tubes filled with argon gas.

**Figure 2:**
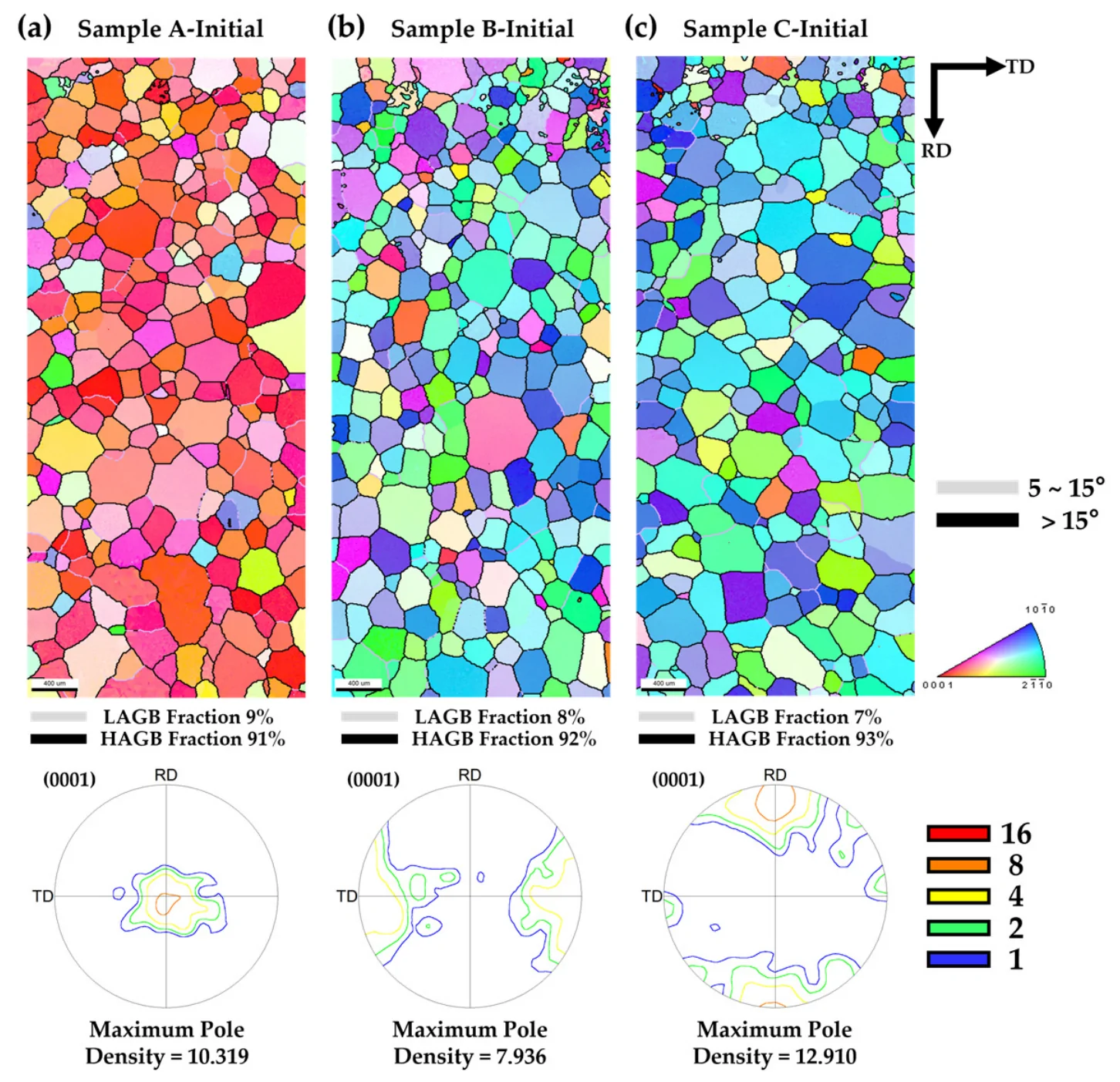
Initial microstructures and (0001) pole figures of Sample A, Sample B, and Sample C specimens after homogenization heat treatment. Pole density contour levels are 1 (blue), 2 (green), 4 (yellow), 8 (orange), and 16 (red), with the same scale used for all contour pole figures.

Figure 2a–c show the microstructures and (0001) pole figures of each sample in the initial condition after homogenization heat treatment before plane strain compression testing. The crystallographic orientation of the microstructures is shown using the standard inverse pole figure (IPF) color code. All microstructure images were analyzed on the compression plane of each Sample. Grain boundaries were classified according to the misorientation angle. Low-angle grain boundaries (LAGB) with misorientation angles of 5–15° are shown in gray. High-angle grain boundaries (HAGB) with misorientation angles higher than 15° are shown in black. A lower misorientation threshold of 5° was adopted for LAGB identification to focus on relatively well-defined subgrain boundaries and to minimize the influence of small local orientation variations. This criterion was applied consistently to all samples to compare the evolution of grain-boundary characteristics under different initial texture conditions. The pole figures show contour maps of the pole density projected onto the analyzed plane.

As shown in Figure 2a–c, Sample A exhibited a strong basal texture with the c-axis preferentially aligned along ND, whereas Samples B and C exhibited initial texture components oriented toward Transverse Direction (TD) and Rolling Direction (RD), respectively. These initial texture orientations are consistent with the specimen configurations schematically illustrated in Figure 1c. These texture features are consistent with the hot rolled microstructures previously reported for AZ80 and AZ91 alloys [8,13,14]. The Mg–Ag alloy showed a basal texture intensity approximately twice as high as that reported for AZ-series Mg alloys [19]. After heat treatment, the initial average grain sizes were measured to be 219 μm for Sample A, 205 μm for Sample B, and 214 μm for Sample C.

High-temperature plane strain compression (PSC) tests were performed using a universal testing machine (Shimadzu Corporation, Kyoto, Japan) equipped with a channel die, as schematically illustrated in Figure 1d. The channel die was employed to constrain deformation along the specimen transverse direction (TD), thereby promoting plane strain deformation primarily in the RD–ND plane. This configuration was selected to minimize lateral deformation along TD and provide a consistent deformation condition for evaluating the effects of initial texture orientation on slip activity, dynamic recrystallization (DRX), and subsequent texture evolution. The specimens were compressed at 723 K and a strain rate of 0.05 s^−1^ to true strains of −0.4, −0.7, and −1.0. After deformation, the specimens were water quenched to prevent additional microstructural changes.

For microstructure and texture analyses, sections were taken from the mid plane parallel to the compression plane. The surfaces for Field-Emission Scanning Electron Microscope (FE-SEM) and Electron Backscatter Diffraction System (EBSD) analyses were mechanically polished up to #4000 grit and then electropolished. The electrolyte was a solution of 3–5% perchloric acid in 99% ethanol. Electropolishing was performed at 243 K. The voltage was adjusted to maintain a current density of 0.19–0.21 A/cm^2^.

Microstructural observations were performed using a field-emission scanning electron microscope (FE-SEM; Sigma 360, Carl Zeiss, Oberkochen, Germany) equipped with an electron backscatter diffraction system (EBSD; Velocity Super, EDAX, AMETEK, Berwyn, PA, USA). EBSD measurements were conducted at an accelerating voltage of 20 kV. A step size of 4 μm was used for large-area microstructural and texture analyses at a magnification of 40×, whereas a finer step size of 0.8 μm was employed over an area of 1200 μm × 1200 μm for detailed microstructural analyses, including KAM and GOS evaluations. The raw data were analyzed using TSL software (OIM Analysis 9). Approximately 200,000 points were examined in each EBSD map, and only datasets with an initial indexing rate exceeding 90% were used for analysis. EBSD data cleanup was performed using the Grain Dilation function in OIM Analysis 9 (EDAX), with a confidence index (CI) threshold of 0.1, to reduce the influence of poorly indexed points on the subsequent microstructural analysis. The average grain size was determined from EBSD data using OIM Analysis 9 (EDAX). Individual grains were identified using a grain-boundary misorientation threshold of 15°, and the average grain size was obtained from the Number average in the Grain Size (diameter) analysis.

## 3. Results

### 3.1. True Stress–Strain Curves

**Figure 3:**
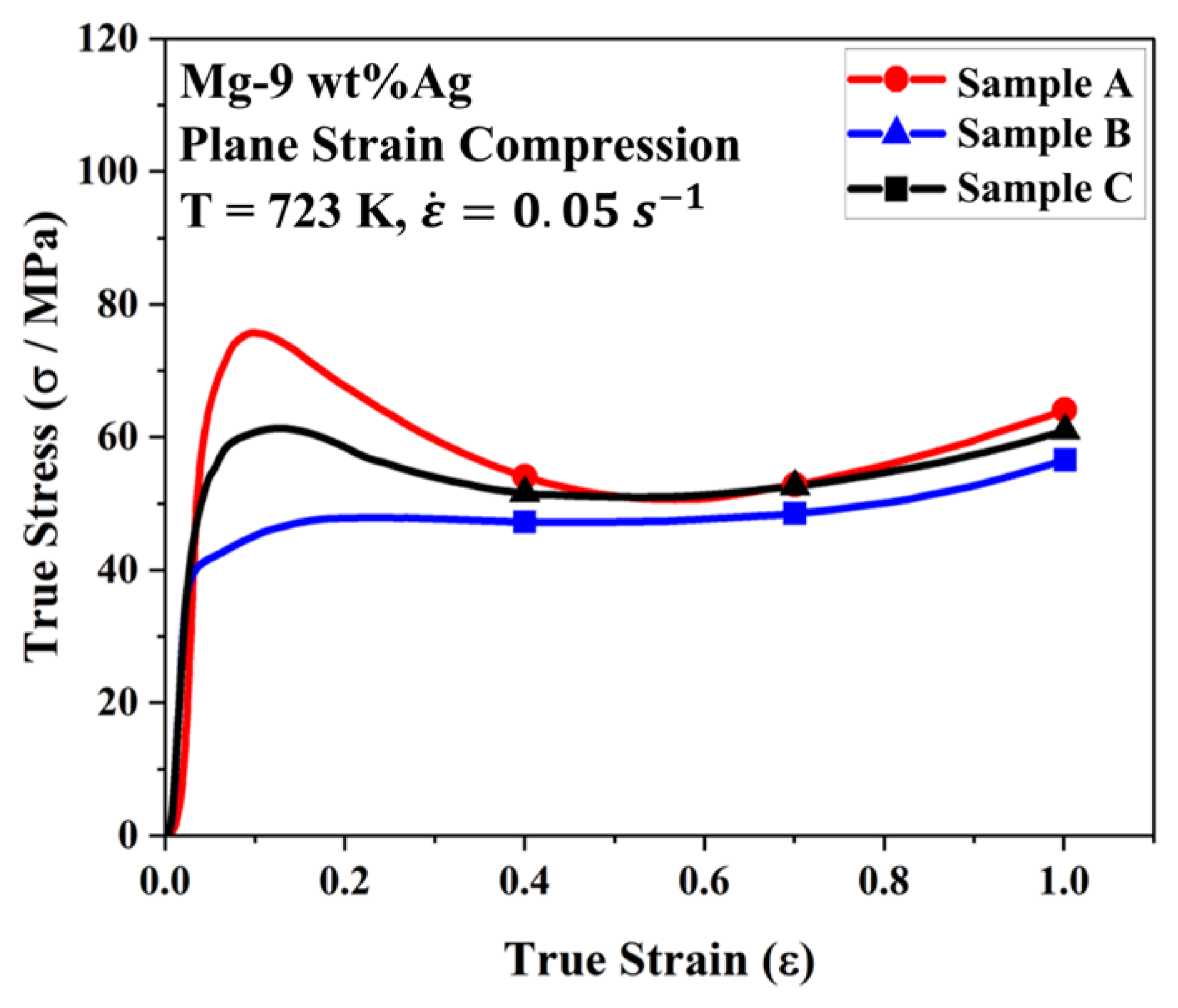
True Stress–Strain Curves of Three Samples of Mg–9 wt.% Ag Alloys Compressed to a Strain of −1.0.

Figure 3 shows the true stress–true strain curves obtained at 723 K and a strain rate of 0.05 s^−1^ up to a true strain of −1.0. The strain values in the figure are presented as absolute values.

For Sample A and Sample C specimens, the stress rapidly increased at the early stage of deformation and then reached a peak stress. After the peak stress, the stress decreased due to work softening. The stress flow after the peak stress showed a steady-state flow behavior. However, at strains higher than −0.7, the flow stress began to increase again. Sample A and Sample C showed similar flow stress characteristics, but some differences were observed depending on the initial texture. Sample A showed the highest peak stress among the three Samples. It also showed the most pronounced change in flow stress. Sample C showed a lower flow stress than Sample A.

Sample B showed the lowest flow stress during the entire deformation process up to a strain of −1.0. In addition, no clear peak stress was observed in Sample B.

The overall shape of the true stress–strain curves was similar to that previously reported for AZ91(MgAl9Zn1) alloys during high-temperature plane strain compression [19]. However, a clear difference was observed under the same specimen preparation conditions. The initial grain size of the Mg–Ag alloy was approximately 200 μm, whereas that of the AZ series alloys was approximately 50 μm. Thus, the Mg–Ag alloy had an average grain size about four times larger than that of the AZ series alloys. Nevertheless, the peak stress of the Mg–Ag alloy was almost twice as high as that of the AZ91 alloy [19]. This high peak stress suggests that the strengthening effect related to Ag addition may have increased the deformation resistance of the Mg–Ag alloy. However, the main focus of this study is not to quantify the effect of Ag addition. Instead, this study focuses on comparing the deformation behavior and texture development caused by differences in the initial texture.

### 3.2. Texture Deformation

#### 3.2.1. Texture Evolution Characteristics of Sample A

**Figure 4:**
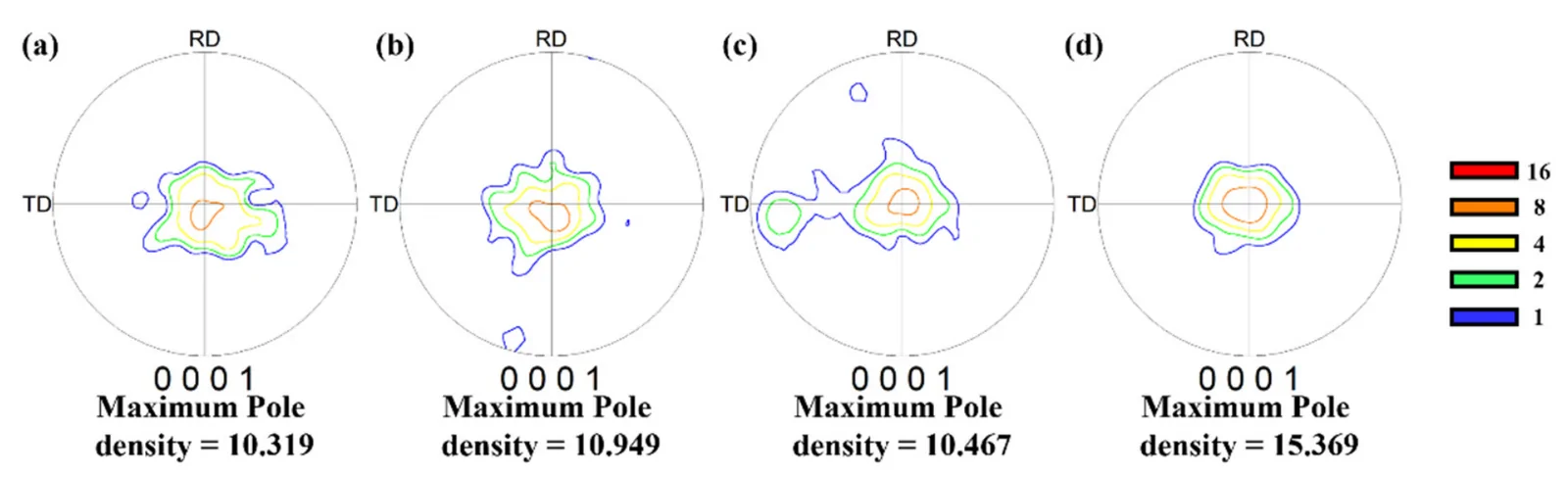
Evolution of (0001) pole figures with increasing true strain in Sample A. True strains: (**a**) 0; (**b**) −0.4; (**c**) −0.7; (**d**) −1.0.

Figure 4 shows the texture changes in Sample A specimens at different strain levels based on the EBSD measurements. In all cases, the pole density was projected onto the compression plane. Before compression, the Sample A specimen showed a basal texture formed during hot rolling. The maximum pole density was 10.319. As plane strain compression proceeded, the pole density gradually increased. A strong (0001) basal texture was maintained even after deformation to −1.0. Compared with the results for the previously reported AZ91 alloy with the same initial texture Sample, the Sample A specimen of the Mg–Ag alloy also maintained its initial texture after deformation to −1.0. In contrast, the maximum pole density after deformation in the AZ-series alloy remained similar to the initial value, whereas that of the Mg–Ag alloy continuously increased with increasing strain [14,19].

#### 3.2.2. Texture Evolution Characteristics of Sample B

**Figure 5:**
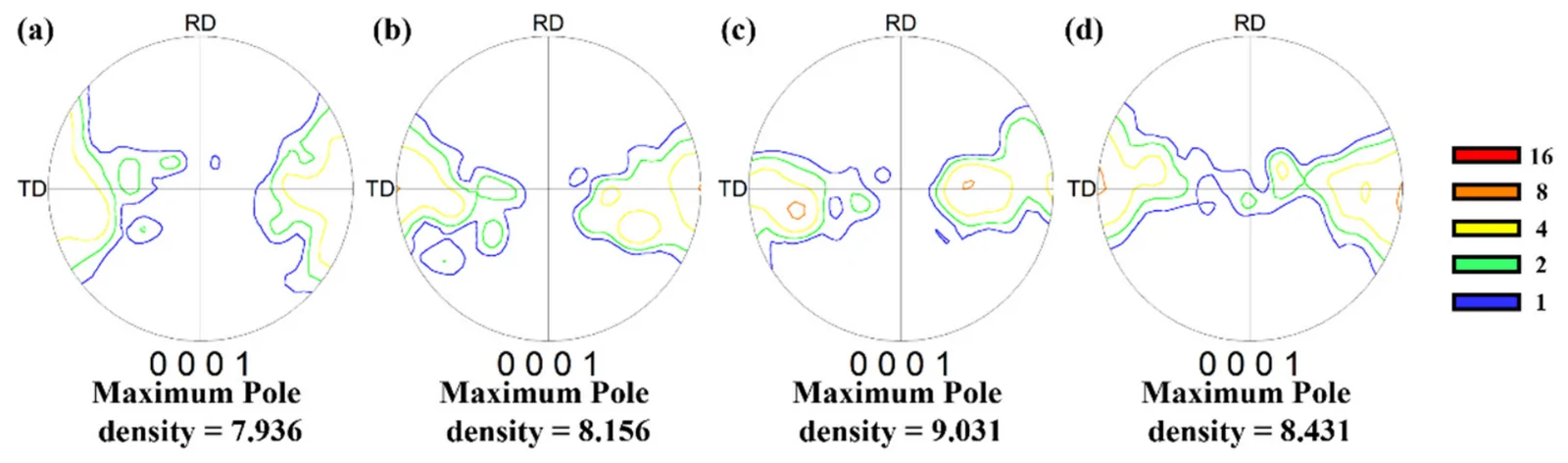
Evolution of (0001) pole figures with increasing true strain in Sample B. True strains: (**a**) 0; (**b**) −0.4; (**c**) −0.7; (**d**) −1.0.

Figure 5 shows the texture changes in Sample B specimens at different strain levels based on the EBSD measurements. Before compression, the Sample B specimen showed a TD-oriented texture. This texture was mostly maintained even after deformation to −1.0. The maximum pole density slightly increased with increasing strain. As deformation proceeded, the maximum pole density gradually shifted toward the ND. However, when the strain reached −1.0, it shifted back toward the TD. Similar to the results reported for AZ alloys, the compressed specimen maintained a strong TD-oriented texture and also developed a partial ND-oriented texture component [13,14,19].

#### 3.2.3. Texture Evolution Characteristics of Sample C

**Figure 6:**
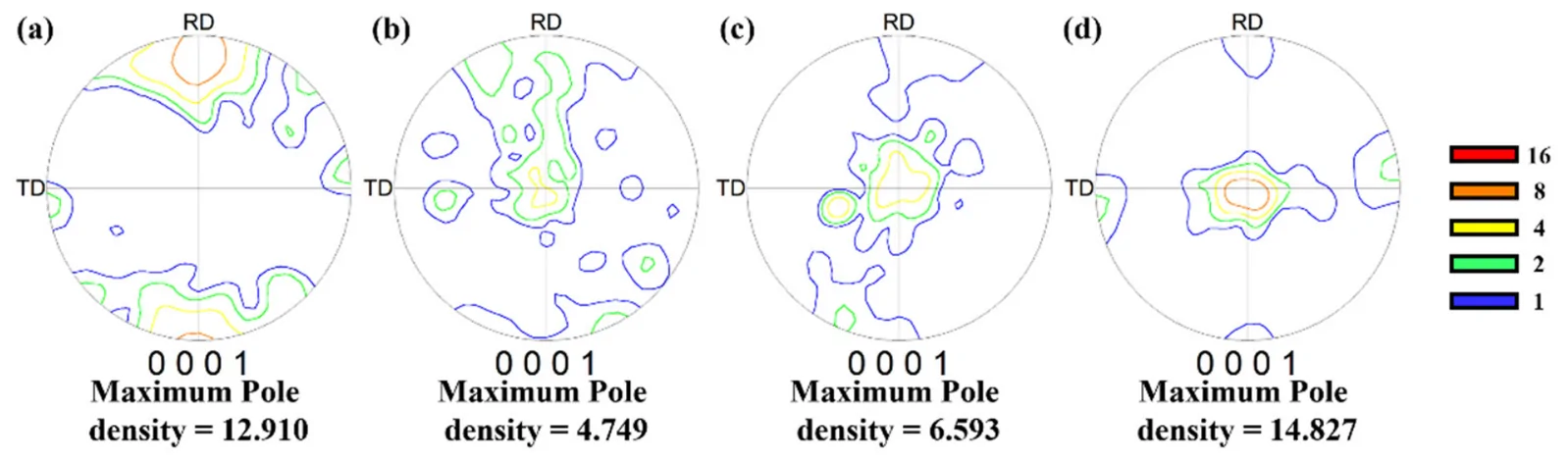
Evolution of (0001) pole figures with increasing true strain in Sample C. True strains: (**a**) 0; (**b**) −0.4; (**c**) −0.7; (**d**) −1.0.

Figure 6 shows the texture changes in Sample C specimens at different strain levels based on the EBSD measurements. Before compression, the Sample C specimen showed an RD-oriented texture formed during hot rolling. As deformation proceeded, the texture gradually shifted toward the ND. A (0001) basal texture developed as the main component, and a small amount of TD-oriented texture was also observed. Unlike the Sample B specimen, the Sample C specimen showed a clear increase in basal pole intensity between strains of −0.7 and −1.0. After deformation to −1.0, the maximum basal pole densities of Sample A and Sample C specimens were 15.369 and 14.827.

The Sample C specimen initially showed an RD-oriented texture. After compression to −1.0, however, it showed a basal texture intensity similar to that of the Sample A specimen, which initially had an ND-oriented texture. This result suggests that the basal texture in the Sample C specimen was rapidly and significantly strengthened during deformation. Compared with the previously reported results for the AZ91 alloy, both specimen Samples C showed the development of a (0001) basal texture. However, the increase in the maximum (0001) pole density was more than three times higher in the Mg–Ag alloy. In all three Samples, basal texture strengthening was observed after deformation to −1.0. The deformation induced increase in pole density was more pronounced in the Mg–Ag alloy than in the AZ alloy.

### 3.3. Microstructure Evolution

**Figure 7:**
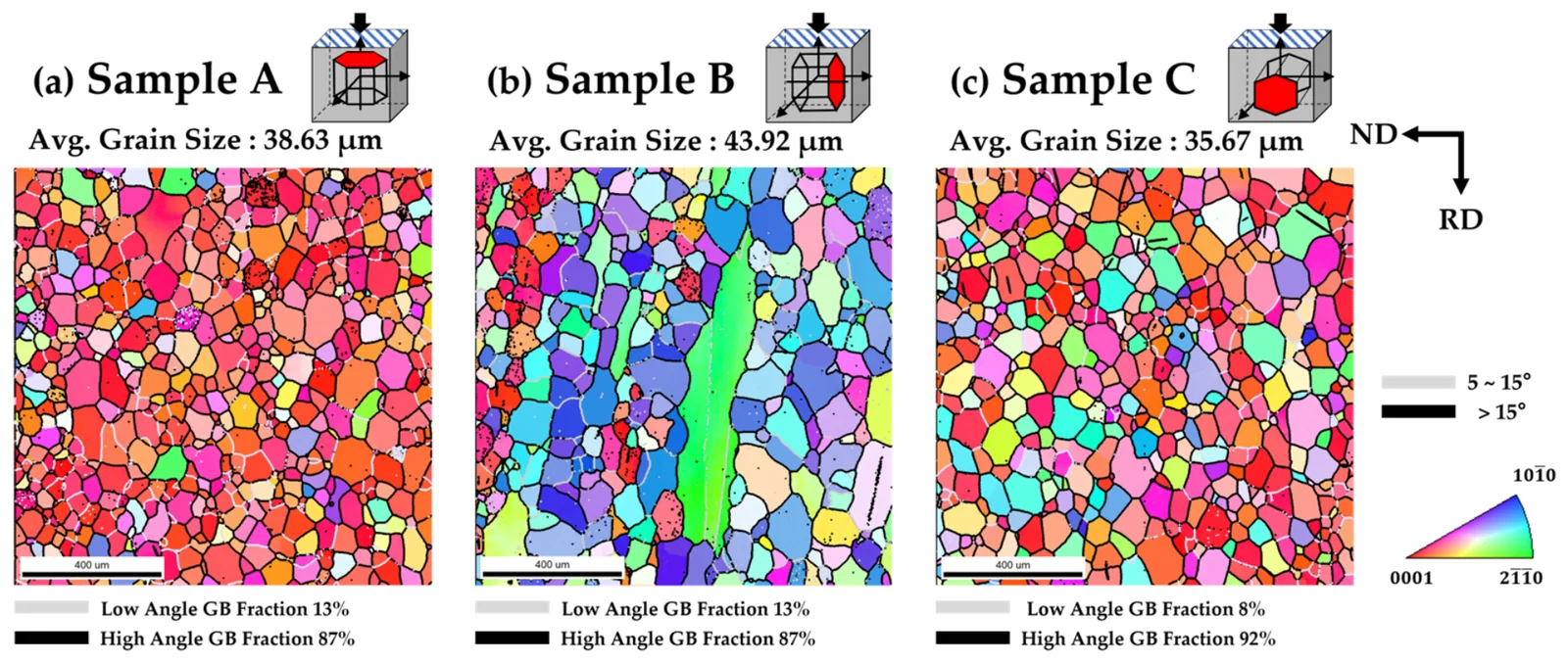
TD (Transverse Direction) Plane Microstructures of Each Sample Based on EBSD Analysis at a Strain of −0.7.

Figure 7 shows the microstructures on the transverse direction (TD) plane of the Mg–9 wt.% Ag alloy specimens after compression to a true strain of −0.7. These microstructures were obtained by SEM-EBSD to more clearly examine the effect of recrystallization behavior on microstructural development. The TD plane is the plane perpendicular to the transverse direction shown in Figure 1. In all three Samples, grain refinement was observed due to dynamic recrystallization (DRX) during deformation. In Sample A and Sample C specimens, equiaxed grains formed by DRX were observed. In contrast, the Sample B specimen was not fully recrystallized. Therefore, relatively coarse grains were intermittently observed in this specimen. These grains contained low-angle grain boundaries (LAGB) aligned vertically along the RD direction. As a result, the Sample B specimen had a larger average grain size than the Sample A and Sample C specimens. In addition, the Sample C specimen showed the lowest LAGB fraction.

**Figure 8:**
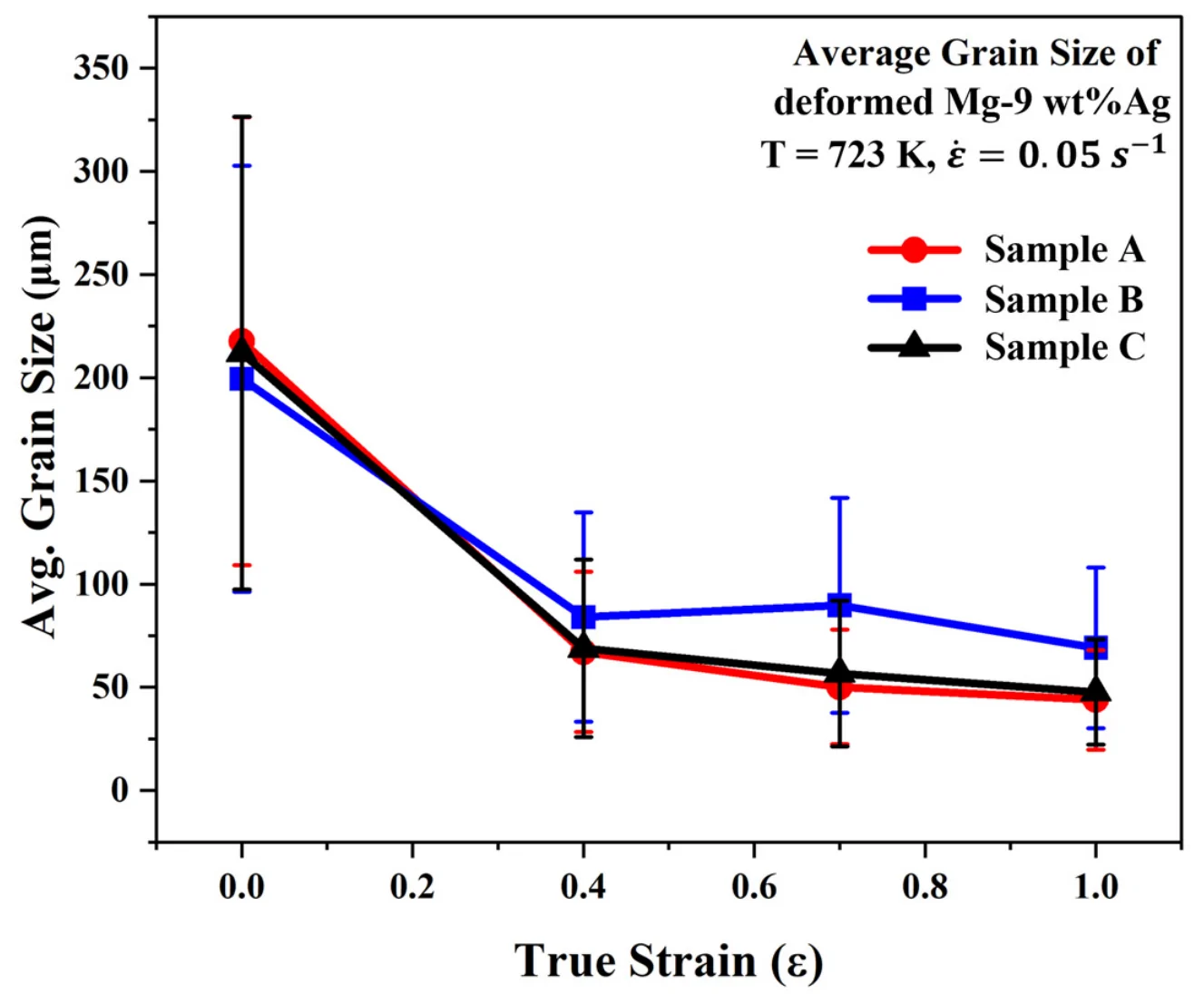
Average Grain Size with Increasing Strain for Each Sample.

Figure 8 shows the average grain size of each sample with increasing strain. Before deformation, the initial average grain size was approximately 200 μm. As the strain increased, the grain size decreased. The standard deviation of the average grain size followed the order of Sample B > Sample C > Sample A. This is considered to be due to the elongated unrecrystallized grains, or non-DRX grains, present in the microstructure. In the Sample B specimen, a rapid decrease in grain size was observed up to a strain of −0.4. After that, the rate of grain size reduction was the lowest among the three samples. Near a strain of −0.7, the grain size slightly increased. This result suggests that the recrystallization behavior was relatively weakened compared with previously reported results for AZ-series alloys under the same deformation conditions [9,13,14,19].

**Figure 9:**
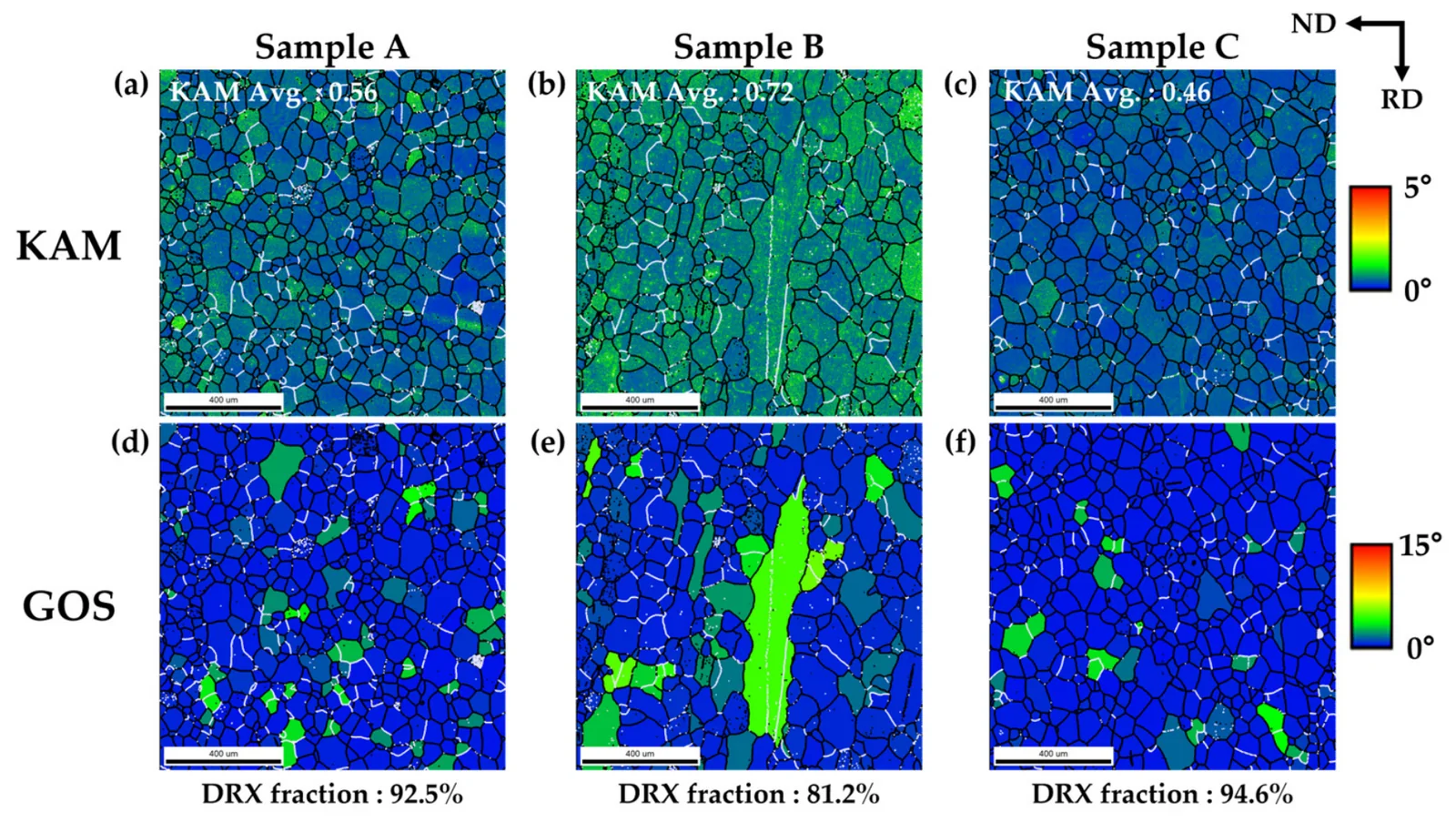
KAM and GOS maps of the Mg–9 wt.% Ag alloy specimens compressed to a true strain of −0.7: (**a**–**c**) KAM maps and (**d**–**f**) GOS maps for Samples A, B, and C.

Figure 9 shows the KAM (kernel average misorientation) and GOS (grain orientation spread) maps of the microstructures on the TD plane for each specimen Sample at a strain of −0.7. The KAM map provides information on the stored energy associated with dislocations and local misorientation inside the grains. The average KAM value followed the order of Sample B > Sample A > Sample C. This indicates that the stored energy inside the grains was highest in Sample B and lowest in Sample C.

In the GOS map, grains with an internal misorientation of 2° or lower are shown in blue, whereas grains with values higher than 2° are shown in green. The blue grains can be regarded as recrystallized grains, while the green grains can be regarded as deformed grains. Based on this classification, the recrystallized fraction followed the order of Sample C > Sample A > Sample B.

These results show that fine equiaxed DRX grains were uniformly distributed in Sample A and Sample C specimens. In contrast, coarse unrecrystallized grains remained in the Sample B specimen. LAGBs aligned along the RD direction were also observed in Sample B. As a result, Sample B showed the highest average KAM value and the lowest recrystallized fraction based on this figure.

## 4. Discussion

### 4.1. Deformation Mechanism

In this study, Mg–9 wt.% Ag alloy specimens with different initial compression planes were conducted to high-temperature plane strain compression. The effects of the initial texture on slip activation, dynamic recrystallization, and final texture development were analyzed. Although all three Samples were deformed at the same temperature and strain rate, they showed different texture evolution and microstructural development depending on the initial texture.

Sample A had a strong basal texture from the initial state, and its maximum pole density continuously increased with increasing strain. Sample B maintained the TD-oriented texture component and also developed a partial ND-oriented component. Sample C rapidly shifted from the initial RD-oriented texture to an ND-oriented basal texture during deformation, and this texture strongly developed.

Finally, a basal texture developed in all three Samples. However, the rate of texture development and the change in pole density depended on the initial texture.

These results are generally consistent with previous studies on AZ-series Mg alloys, which showed that the initial texture controls texture evolution during high-temperature plane strain compression [13,14,19]. In studies on the plane strain compression of AZ31B and AZ80 alloys at 723 K, various stable or metastable texture components were formed depending on the initial texture. It was also reported that the $\left\{11\bar{2}0\right\}<10\bar{1}0>$ texture developed when non-basal slip was dominant [12]. In addition, at high temperatures, the difference in CRSS between basal and non-basal slip decreases. Therefore, the relative contributions of basal, prismatic, and pyramidal slip can change depending on the initial texture [10,11].

Compared with AZ series alloys deformed under the same conditions, previous studies on the high-temperature plane strain compression of AZ80 and AZ91 alloys reported that stable orientation components related to basal, prismatic, and pyramidal slip systems were formed depending on the initial texture. These studies also showed that basal texture components finally developed after deformation. In particular, in the AZ80 alloy, $\left(0001\right)<10\bar{1}0>,\;\left(0001\right)<11\bar{2}0>,\;\left\{11\bar{2}0\right\}<10\bar{1}0>,\;\{10\bar{1}0\}<0001>$ components appeared depending on the initial texture. These components were analyzed as stable orientations under plane strain compression conditions [13,14,19]. In Mg–Ag alloy, basal texture finally developed in all three Samples. This behavior is similar to that observed in AZ series alloys. However, the Mg–Ag alloy showed a higher flow stress and a stronger increase in basal pole density than the reported AZ series alloys. However, the three samples had the same chemical composition and were deformed under the same conditions. Therefore, the differences among the specimen Samples can be mainly interpreted in terms of differences in slip activity and lattice rotation induced by the initial texture.

As reported by Kim et al. [9], deformation of conventional Mg alloys should be accommodated by at least five independent slip systems according to the Von Mises theory. Slip systems other than basal slip have high CRSS values and are difficult to activate at room temperature. Therefore, Mg alloys often show poor formability. When the number of active slip systems is not sufficient during deformation, twinning deformation, such as tensile twinning, is generally also involved.

However, when deformation is carried out at relatively high temperatures, the CRSS values of non-basal and basal slip systems become similar above approximately 400 °C. In this temperature range, the activation of non-basal slip systems can satisfy the Von Mises theory. As a result, the contribution of twinning deformation during deformation can be reduced [10,11].

**Figure 10:**
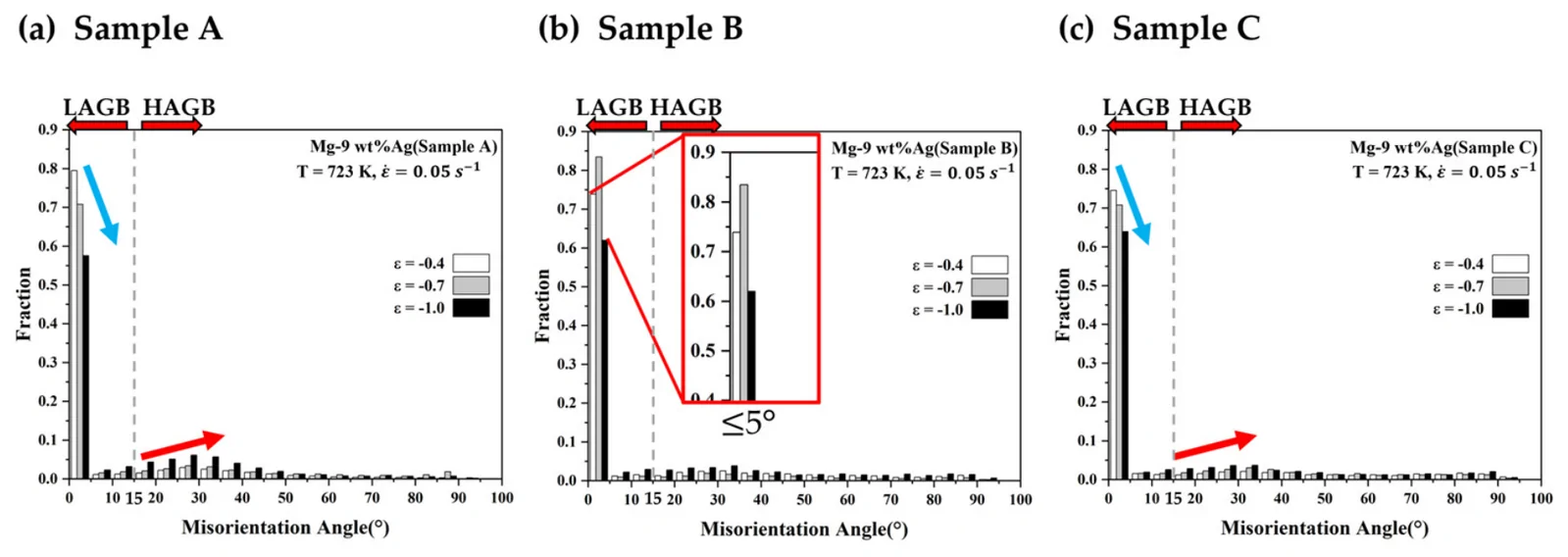
Misorientation Angle Fraction for Each Specimen Sample.

In this study, as shown in Figure 10, increase was not observed in the fractions of the 56° and 86° misorientation angles, which are commonly associated with twinning in Mg alloys. Therefore, the possibility that twinning contributed to the observed texture evolution was considered to be limited under the present deformation condition.

In addition, as compression proceeded, the fraction of LAGB with misorientation angles lower than 15° decreased, while the fraction of HAGBs with misorientation angles higher than 15° increased. This change in the misorientation angle fraction is known to be a typical behavior during DRX. It is also consistent with the steady-state region observed in the stress–strain curves. However, in the Sample B specimen, a slight increase in the LAGB fraction was observed near a strain of −0.7. This increase is expected to be related to dislocation rearrangement inside the grains by DRV. It is less likely to be caused by the consumption of low-angle grain boundaries below 5° during DRX. This is also consistent with the KAM and GOS map results shown in Figure 9. Therefore, the difference in texture development among the specimen samples was mainly attributed to the combined effects of slip and recrystallization behavior caused by differences in the initial texture, rather than the effect of twinning.

### 4.2. Microstructure Development

#### 4.2.1. Slip Behavior Depending on the Initial Texture

Under the same deformation conditions, the difference in texture development among the specimen samples can be mainly understood from the relationship between slip and recrystallization caused by the initial texture. Among these factors, slip behavior can vary depending on the geometrical relationship of the initial texture [9]. To examine the effect of the initial texture on slip behavior in each specimen sample, the activation tendency was evaluated based on the Schmid factor equation.

$$\tau_{\mathrm{RSS}}=\sigma \cos\lambda \cos\Phi$$

**Figure 11:**
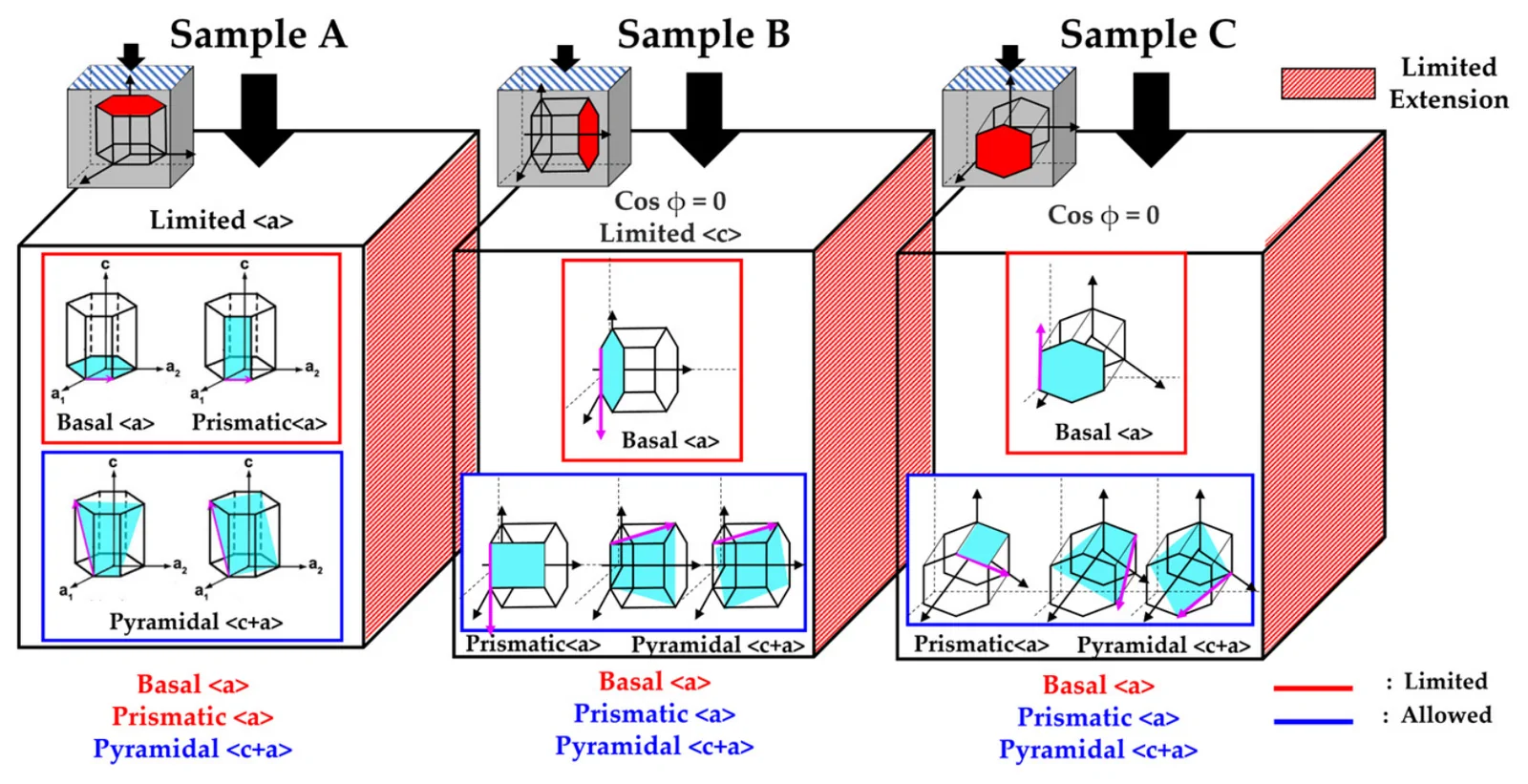
Schematic illustration of the geometrical relationship between the initial crystallographic orientation and potential slip systems under plane strain compression.

In this equation, λ and Φ represent the angles related to the compression axis, the slip direction, and the slip plane normal. When the c-axis of a grain is perpendicular to the compression axis, slip activation can be difficult. This corresponds to the case where the (0001) basal plane is perpendicular to the compression axis. Based on this geometrical relationship, the slip systems that are theoretically difficult to activate for each initial texture sample are shown in Figure 11. However, this analysis is based on an ideal geometrical relationship, in which the relevant directions are exactly perpendicular to each other. Therefore, the actual primary active slip systems may be different from those predicted from this simple geometrical consideration.

To identify the primary active slip systems depending on the initial texture, the average Schmid factor values were calculated from the EBSD data. Based on these values, the primary active slip systems for each specimen sample were summarized, as shown in Table 2.

Schmid factor calculation using EBSD orientation data is a useful method to evaluate whether a specific slip system is geometrically favorable for activation in each grain. However, the Schmid factor does not directly show the actual active slip system. It only represents the geometrical term of the resolved shear stress. Therefore, it should be analyzed together with the CRSS of each slip system. In Mg alloys, basal slip generally has the lowest CRSS. However, the CRSS values of prismatic and pyramidal slip decrease at high temperatures. As a result, the relative difference between basal and non-basal slip becomes smaller. Therefore, the slip systems with high Schmid factor values were not directly regarded as the primary active slip systems. Instead, they were interpreted as slip systems that are more likely to be preferentially activated under the present deformation condition.

In the Sample A specimen, the basal poles were concentrated near the center of the compression plane from the initial state. As deformation proceeded, the basal pole density continuously increased. This indicates that the basal texture component worked as a stable orientation during plane strain compression in Sample A. Although the Schmid factor for basal slip can be low in an ideal basal orientation, the actual EBSD map includes tilted grains that are not perfectly aligned with the ideal orientation. Therefore, basal slip can be locally activated in these grains. This local basal slip and the following lattice rotation are considered to have contributed to the increase in the final basal texture intensity.

In the Sample B specimen, the initial c-axis had an orientation component related to the TD. The Schmid factor analysis also showed a high possibility of prismatic slip activation at the early stage of deformation. Previous studies on the plane strain compression of AZ80 alloys reported that prismatic slip can suppress the rapid development of basal texture. It can also contribute to the retention or development of a prismatic texture in which the c-axis is parallel to the TD [8]. In this study, the Sample B specimen also maintained a considerable TD-oriented component after deformation. In addition, the increase in pole density was more limited than those in Sample A and Sample C specimens. Therefore, in Sample B, prismatic slip is considered to have maintained the metastable texture component and delayed rapid rotation toward the stable basal component.

In the Sample C specimen, prismatic slip was geometrically favorable in the initial state. However, as deformation proceeded, the texture rapidly changed toward a condition where basal slip became dominant. In particular, the sharp increase in basal pole density between −0.7 and −1.0 indicates that the initial RD-oriented component in Sample C rapidly rotated toward the stable basal texture component. It has been reported that a dominant pyramidal <c+a> slip can cause tilting of the basal poles toward the RD. However, in this study, a clear RD tilted basal texture was not observed as the final main component. Therefore, pyramidal slip may have operated locally. However, it is difficult to regard it as the main deformation mode controlling the overall texture evolution under the this conditions. Previous studies on low strain rate high-temperature plane strain compression of AZ80 alloys reported that prismatic and pyramidal slip can suppress basal texture development or induce an approximately 20° tilt toward the RD [8,20]. In contrast, the results suggest that the relative contribution of basal/prismatic slip was more dominant.

#### 4.2.2. DRX Mechanism and Recrystallization Behavior Depending on the Initial Texture

**Figure 12:**
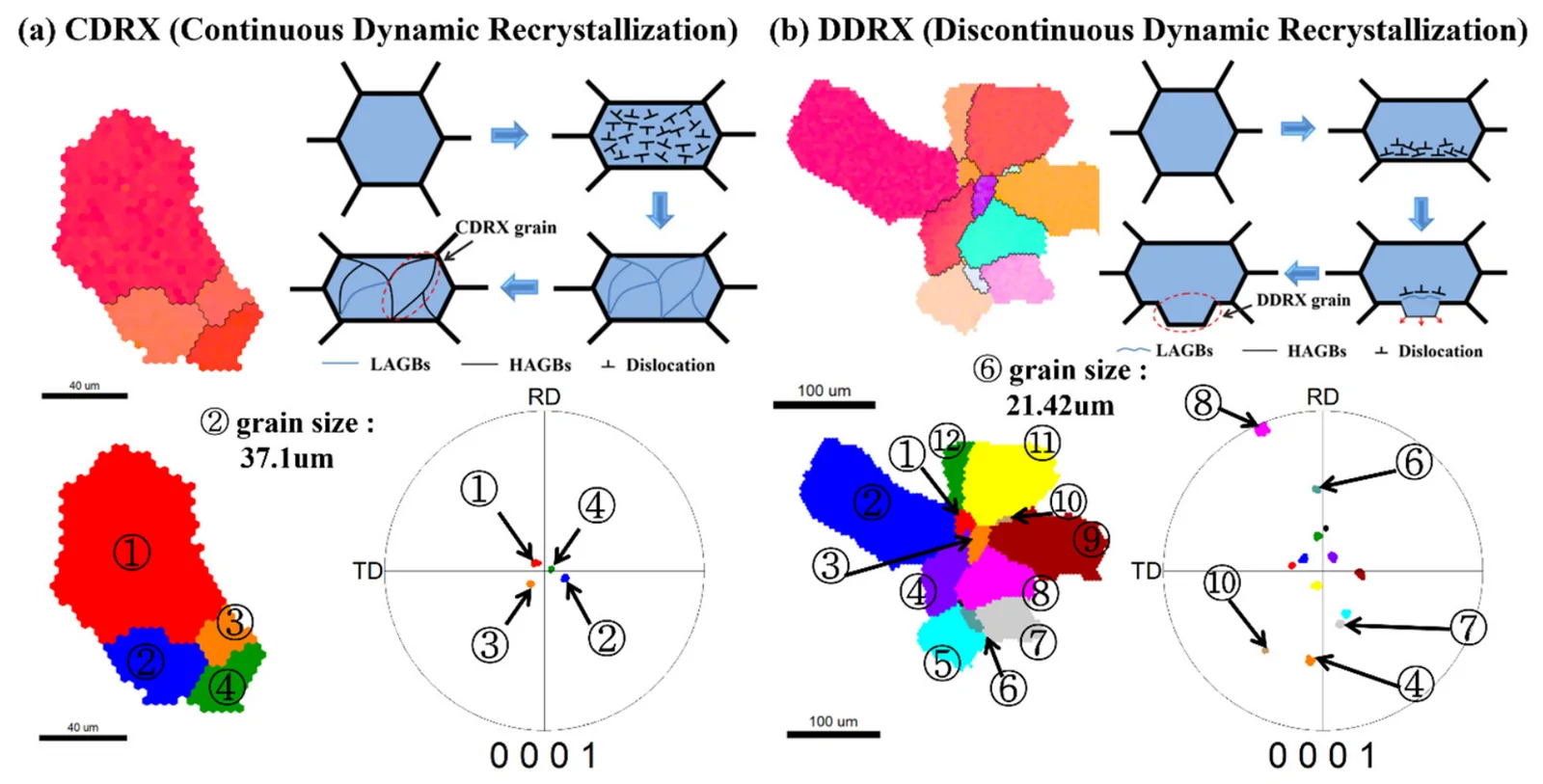
Primary DRX mechanism CDRX & DDRX.

Figure 12 schematically illustrates the mechanisms of continuous dynamic recrystallization (CDRX) and discontinuous dynamic recrystallization (DDRX). Following the discussion of slip behavior, the DRX mechanisms used to explain recrystallization behavior can be mainly classified into two samples. When dislocations generated during deformation are distributed inside the grains, subgrain boundaries can form by dislocation rearrangement. These subgrain boundaries gradually rotate and develop into new recrystallized grains. This mechanism is called continuous dynamic recrystallization (CDRX). In CDRX, the recrystallized grains form by gradual rotation inside the parent grains. Therefore, they tend to inherit the orientation of the parent grains and usually have relatively large grain sizes. In discontinuous dynamic recrystallization (DDRX), dislocations generated during deformation are locally accumulated near grain boundaries. The stored dislocation energy can then drive grain boundary bulging. New recrystallized grains form in the bulged regions. These DDRX grains usually have more random orientations and are relatively smaller than CDRX grains. Therefore, the DRX mechanism can be classified into CDRX and DDRX. The main difference is that CDRX is associated with orientation inheritance and relatively large recrystallized grains, whereas DDRX is associated with random orientations and relatively small recrystallized grains.

The difference in microstructure development can be explained by the combined effects of slip activity and the DRX mechanism. During high-temperature deformation of Mg and Mg alloys, CDRX and DDRX generally operate together. Their relative contributions depend on temperature, strain, strain rate, and the active slip systems. Previous studies on high-temperature compression of Mg also reported that CDRX occurs as the main recrystallization mechanism in the temperature range of 523–773 K. At 723 K, DDRX can also occur through bulging of coarse grain boundaries [21,22].

In this study, fine equiaxed DRX grains were uniformly distributed in Sample A and Sample C specimens. In contrast, coarse unrecrystallized grains and LAGBs aligned along the RD remained in the Sample B specimen. In addition, the average KAM value was highest in Sample B, while the recrystallized fraction based on the GOS map data was lowest in Sample B.

These results suggest that prismatic slip was mainly activated in Sample B. As a result, dislocations were widely distributed inside the grains. These dislocations were then rearranged into subgrain boundaries or LAGB, indicating a strong tendency for DRV/CDRX. Therefore, dynamic recovery and gradual misorientation increase were dominant, rather than rapid DDRX caused by local grain boundary bulging. As a result, coarse non-DRX grains remained, and the average grain size was maintained at a relatively large value.

In contrast, in Sample A and Sample C specimens, basal slip was dominant or rapid lattice rotation toward the basal texture component occurred. In this case, deformation energy may have been concentrated near specific grain boundaries. This condition can promote DDRX through grain boundary bulging. The newly formed fine DDRX grains may have orientations partly different from those of the parent grains. In fact, previous studies on AZ80 alloys reported that DRX grains with relatively random orientations can form by DDRX. These grains can contribute to texture weakening [23].

From this point, the formation of some fine grains with different orientations in Sample A and Sample C can be explained, even though the basal texture was maintained as a stable orientation. However, in this study, the difference in pole figures between DRX grains and non-DRX grains was not large. Therefore, it is reasonable to consider that the overall texture evolution was mainly controlled by slip induced lattice rotation, rather than orientation randomization caused by DRX.

**Figure 13:**
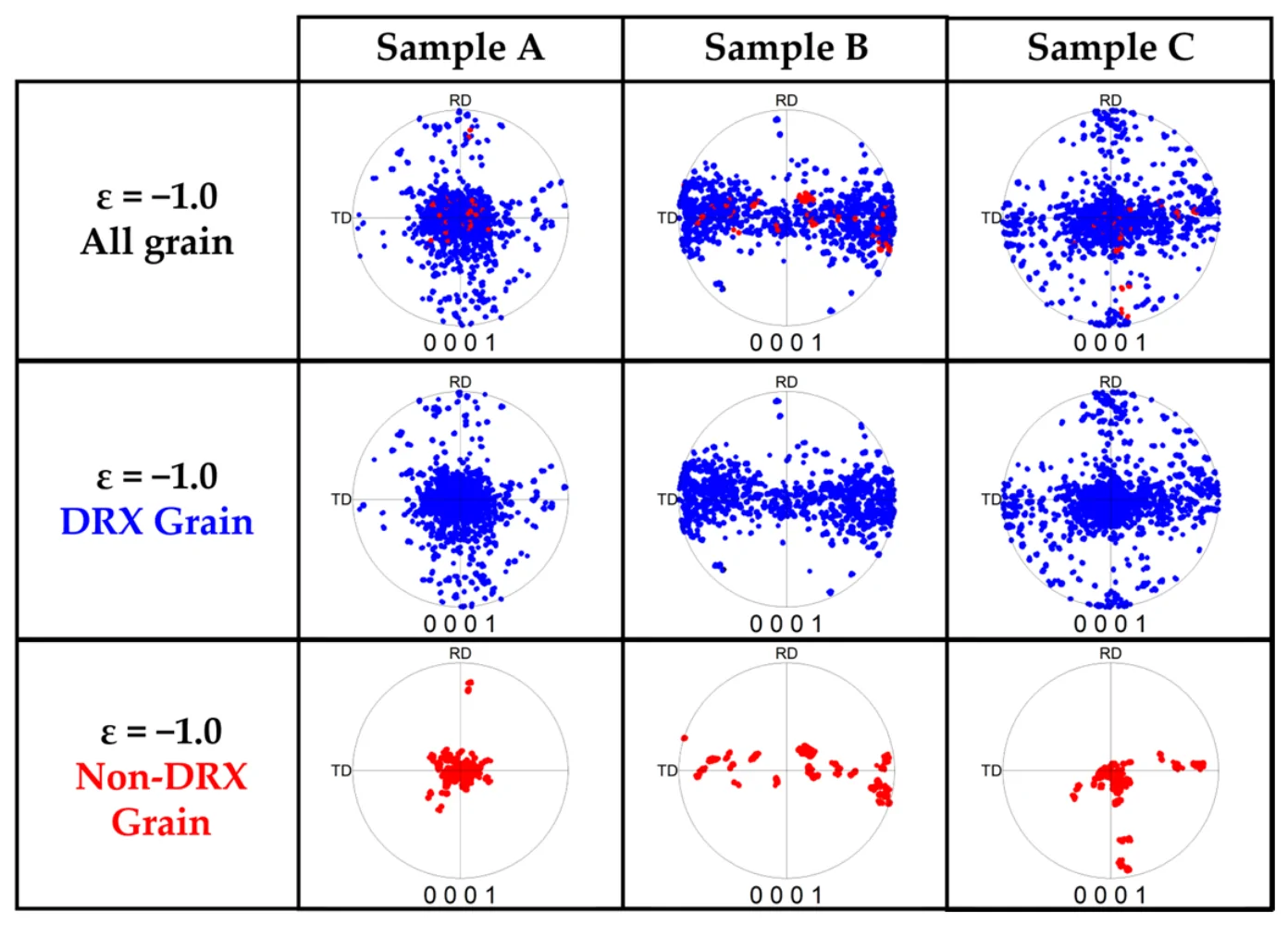
Pole figures of DRX and non-DRX grains separated based on the GOS.

Figure 13 shows the pole figures of DRX and non-DRX grains for each specimen Sample. The grains were classified based on the GOS. Grains with an internal misorientation of 2° or lower were defined as DRX grains, while grains with an internal misorientation higher than 2° were defined as non-DRX grains. As shown in Figure 13, there was no clear texture difference between the DRX and non-DRX grains in all three Samples. Texture weakening could be expected if DDRX occurred significantly. However, this result suggests that the area fraction of CDRX was higher than that of DDRX. Therefore, under the present deformation conditions of the Mg–Ag alloy, recrystallization occurred through a combined mechanism of CDRX and DDRX, but CDRX was relatively dominant. In addition, no clear change in texture components caused by DRX was observed. Overall, the microstructural development in this study was mainly affected by differences in slip behavior caused by the initial texture, as reported in previous studies. These differences may have also caused differences in recrystallization behavior. However, the effect of recrystallization behavior on texture development was limited.

Finally, texture evolution during high-temperature plane strain compression of the Mg–9 wt.% Ag alloy was primarily controlled by the relative activity of basal and prismatic slip, which was determined by the initial texture. In Sample A, the initial basal texture was maintained as a stable component, and the pole density increased with deformation. In Sample B, the TD/prismatic component was maintained as a metastable component due to the contribution of prismatic slip. As a result, the rapid transition toward basal texture was delayed. In Sample C, prismatic slip was possible at the early stage of deformation. However, as deformation proceeded, basal slip and lattice rotation toward the stable basal component became dominant. Finally, a strong basal texture similar to that of Sample A was formed. Therefore, controlling the initial texture and deformation behavior is essential for controlling the final texture of Mg–Ag alloys during hot working.

## 5. Conclusions

The microstructural and textural evolution of a Mg–9 wt.% Ag magnesium alloy was investigated after plane strain compression at 723 K and a strain rate of 5.0 × 10^−2^ s^−1^ up to a true strain of −1.0.

The major results are as follows:

(1) Dynamic recrystallization occurred in all three samples, resulting in significant grain refinement from the initial grain size of approximately 200 μm. Samples A and C exhibited relatively uniform distributions of fine equiaxed dynamically recrystallized grains, whereas Sample B retained coarse unrecrystallized grains with RD-aligned low-angle grain boundaries.

(2) The dominant slip activity was strongly affected by the initial texture. Although basal slip has the lowest CRSS in Mg alloys, the reduced CRSS gap between basal and non-basal slip systems at 723 K allowed prismatic slip to contribute significantly, particularly in Sample B during the early stage of deformation.

(3) Sample B exhibited the highest KAM value, the lowest DRX fraction, and retained coarse non-recrystallized grains. These features suggest that prismatic slip promoted a more homogeneous distribution and rearrangement of dislocations, leading to enhanced DRV/CDRX behavior and delayed recrystallization.

(4) Although dynamic recrystallization contributed to grain refinement and local orientation scattering, the final texture evolution was governed mainly by initial texture dependent slip induced lattice rotation. These results demonstrate that the initial texture and the associated basal and prismatic slip tendency are the primary factors controlling basal texture development in the Mg–9 wt.% Ag alloy in this study plane strain compression condition.

## Figures and Tables

**Table 1:** Chemical composition of experiment alloy (wt.%).

	Ag	Al	Zn	Mn	Mg
Mg–9 wt.% Ag	9.27	0.04	0.01	0.01	Bal.

**Table 2:** Slip systems with the highest average Schmid factor for each specimen sample.

Sample	0	−0.4	−0.7
ε			
Sample A	Basal	Basal	Basal
Sample B	Prismatic	Prismatic	Basal
Sample C	Prismatic	Basal	Basal

## Data Availability

The original contributions presented in this study are included in the article. Further inquiries can be directed to the corresponding authors.

## Abbreviations

The following abbreviations are used in this manuscript:

PSC	Plane Strain Compression
CRSS	Critical Resolved Shear Stress
CDRX	Continuous Dynamic Recrystallization
DDRX	Discontinuous Dynamic Recrystallization
LAGB	Low-Angle Grain Boundary
HAGB	High-Angle Grain Boundary

## References

[1] Jiang Z., Hu B., Li Z., Yao F., Han J., Li D., Zeng X., Ding W. (2024). A review of magnesium alloys as structure–function integrated materials. Acta Metall. Sin. (Engl. Lett.).

[2] Yemata T.A., Lu H., Zheng Y., Kyaw A.K.K., Zhu Q., Xu J., Chin W.S., Hayashi Y., Tadsual M.K. (2026). Biodegradable magnesium alloys and magnesium metal matrix composites (Mg/MMCs) in biomedical applications: A comprehensive state-of-the-art review based on materials, processing techniques, design, and application perspective. Emergent Mater..

[3] Nie J.F., Shin K.S., Zeng Z.R. (2020). Microstructure, deformation, and property of wrought magnesium alloys. Metall. Mater. Trans. A.

[4] Bai J., Yang Y., Wen C., Chen J., Zhou G., Jiang B., Peng X., Pan F. (2023). Applications of magnesium alloys for aerospace: A review. J. Magnes. Alloys.

[5] Liu B., Yang J., Zhang X., Yang Q., Zhang J., Li X. (2023). Development and application of magnesium alloy parts for automotive OEMs: A review. J. Magnes. Alloys.

[6] Ramya M. (2025). Advances in biodegradable orthopaedic implants: Optimizing magnesium alloy corrosion resistance for enhanced bone repair. Biomed. Mater. Devices.

[7] Tsakiris V., Tardei C., Clicinschi F.M. (2021). Biodegradable Mg alloys for orthopedic implants: A review. J. Magnes. Alloys.

[8] Ji Y., Yun J., Kim K., Lee T.H., Kim K. (2024). The influence of the strain rate on texture formation during the plane strain compression of AZ80 magnesium alloy. Materials.

[9] Kim K., Ji Y., Kim K., Park M. (2023). Effect of Al concentration on basal texture formation behavior of AZ-series magnesium alloys during high-temperature deformation. Materials.

[10] Shin K.S., Wang L., Bian M., Choi S., Komissarov A., Bazhenov V. (2023). Effects of temperature on critical resolved shear stresses of slip and twining in Mg single crystal via experimental and crystal plasticity modeling. J. Magnes. Alloys.

[11] Barnett M.R. (2003). A Taylor model based description of the proof stress of magnesium AZ31 during hot working. Metall. Mater. Trans. A.

[12] Fukutomi H., Park H.G., Kim K.H., Okayasu K., Hasegawa M., Umezawa O. (2019). Mechanism of texture and microstructure evolution during high temperature plane strain compression in AZ31B and AZ80 magnesium alloys with various initial textures. J. Jpn. Inst. Met. Mater..

[13] Kim K.H., Okayasu K., Fukutomi H. (2015). Influence of the initial texture on texture formation of high temperature deformation in AZ80 magnesium alloy. Mater. Trans..

[14] Kim J., Okayasu K., Fukutomi H. (2012). Texture evolution in AZ80 magnesium alloy by the plane strain compression deformation at high temperature. Mater. Trans..

[15] Liu G., Wang K., Pan Y., Yang D., Yu X., Nie Z., Zhu J., Han J., Tan C. (2024). Effect of Ag content and extrusion on the microstructure and mechanical properties of Mg–Ag alloys. J. Mater. Res. Technol..

[16] Ouyang S., Wu X., Meng L., Jing X., Qiao L., She J., Zheng K., Chen X., Pan F. (2024). More than the barrier effect: Biodegradable Mg–Ag alloy membranes for guided bone/tissue regeneration. J. Magnes. Alloys.

[17] Liu G., Han J., Yu X., Yuan S., Nie Z., Qiu T., Yan Z., Tan C., Guo C. (2022). Influences of extrusion and silver content on the degradation of Mg–Ag alloys in vitro and in vivo. Bioinorg. Chem. Appl..

[18] Tie D., Feyerabend F., Müller W.-D., Schade R., Liefeith K., Kainer K.U., Willumeit R. (2013). Antibacterial biodegradable Mg–Ag alloys. Eur. Cells Mater..

[19] Han D. (2020). Effect of Initial Texture and Deformation Condition on Texture of AZ91 Magnesium Alloy During High Temperature. Master’s Thesis.

[20] Ji Y.B., Lee J.H., Kim K.H. (2022). The effect of strain rate on the texture formation behavior of AZ80 magnesium during high-temperature deformation. MRS Adv..

[21] Sitdikov O., Kaibyshev R. (2001). Dynamic recrystallization in pure magnesium. Mater. Trans..

[22] Wang T., Jonas J.J., Yue S. (2017). Dynamic recrystallization behavior of a coarse-grained Mg-2Zn-2Nd magnesium alloy. Metall. Mater. Trans. A.

[23] Qin D.H., Wang M.J., Sun C.Y., Su Z.X., Qian L.Y., Sun Z.H. (2020). Interaction between texture evolution and dynamic recrystallization of extruded AZ80 magnesium alloy during hot deformation. Mater. Sci. Eng. A.

